# Older people coping with low mood: a qualitative study

**DOI:** 10.1017/S1041610215002264

**Published:** 2015-12-17

**Authors:** Margaret von Faber, Geertje van der Geest, Gerda M. van der Weele, Jeanet W. Blom, Roos C. van der Mast, Ria Reis, Jacobijn Gussekloo

**Affiliations:** 1 Department of Public Health and Primary Care, Leiden University Medical Center, PO Box 9600, 2300 RC Leiden, the Netherlands; 2 Department of Guideline Development and Research, Dutch College of General Practitioners, PO Box 3231, 3502 GE Utrecht, the Netherlands; 3 Department of Psychiatry, Leiden University Medical Center PO Box 9600, 2300 RC Leiden, the Netherlands; 4 Department of Psychiatry, CAPRI-University of Antwerp, 2610 Antwerp, Belgium; 5 Amsterdam Institute for Social Science Research, University of Amsterdam PO Box 15718, 1001 NE Amsterdam, the Netherlands; 6 The Children's Institute, University of Cape Town, Cape Town, South Africa

**Keywords:** qualitative research, older adults, depressive symptoms, coping, the Netherlands

## Abstract

**Background:**

To gain new insight into support for older people with low mood, the perceptions, strategies, and needs of older people with depressive symptoms were explored.

**Methods:**

Two in-depth interviews were held with 38 participants (aged ≥77 years) who screened positive for depressive symptoms in general practice. To investigate the influence of the presence of complex health problems, 19 persons with and 19 without complex problems were included. Complex problems were defined as a combination of functional, somatic, psychological or social problems.

**Results:**

All participants used several cognitive, social or practical coping strategies. Four patterns emerged: mastery, acceptance, ambivalence, and need for support. Most participants felt they could deal with their feelings sufficiently, whereas a few participants with complex problems expressed a need for professional support. Some participants, especially those with complex problems, were ambivalent about possible interventions mainly because they feared putting their fragile balance at risk due to changes instigated by an intervention.

**Conclusion:**

Most older participants with depressive symptoms perceived their coping strategies to be sufficient. The general practitioners (GPs) can support self-management by talking about the (effectiveness of) personal coping strategies, elaborating on perceptions of risks, providing information, and discussing alternative options with older persons.

## Introduction

Depressive feelings are common at old age and have a negative influence on the health and well-being of older people and their social relations (Beekman *et al.*, 2002; Cuijpers *et al.*, 2009). The prognosis of late-life depression is often poor (Comijs *et al.*, 2011) with a more chronic course and higher relapse rates than depression earlier in life (Stek *et al.*, 2004; Mitchel *et al.*, 2011).

Depressive symptoms at old age are reported to be both under-recognized and under-treated. Therefore, from a professional perspective, screening is suggested to be effective for early detection of depressive symptoms to minimize the risk of developing a major depression by offering interventions targeted at the different aspects and causes of depression (Romijn *et al.*, 2007; Lyness *et al.*, 2009). However, in our earlier combined screening/intervention program, the majority of older people who screened positive for depressive symptoms declined the intervention offered (van der Weele *et al.*, 2012).

Help-seeking behavior of patients is known to be related to their illness experience and perceived needs. Various studies have described the different views of older patients and physicians on the diagnosis of depression and the treatment of depressive symptoms (van Schaik *et al.*, 2004; Prins *et al.*, 2008; Wittkampf *et al.*, 2008). When older persons perceive social and contextual factors as a cause of their distress, they may not label their emotional distress as depression and disease according to the biomedical model. Subsequently, they may have a different viewpoint as to which interventions may be helpful for them, compared with caregivers or healthcare professionals (Beljouw *et al.*, 2014). Older persons prefer psychological treatment to medical treatment and may not want professional treatment unless depression is severe (Joo *et al.*, 2011).

There is evidence that both GPs and older patients may perceive depression as understandable and justifiable in old age (Murray *et al.*, 2006). This may lead to a passive attitude on the part of both older patients and their physicians, as well as patients’ reluctance to present depressive symptoms because of their low expectations of treatment (Burroughs *et al.*, 2006). However, studies also describe perceptions of self-reliance (Prins *et al.*, 2011) self-responsibility and culturally-appropriate coping strategies of older people to combat depressive feelings (Lawrence *et al.*, 2006; Conner *et al.*, 2010). Therefore, it is essential to establish whether older persons expect professional support when they indicate depressive symptoms in screening and how they perceive their own role in dealing with depressive symptoms.

The aim of the qualitative study EPISODE (perceptions of Elderly Patients Interviewed after Screening On Depressive feelings) was to describe the perceptions, coping strategies, and needs of older patients (with and without complex problems) in general practice who screened positive for depressive symptoms. In line with the Dutch College of GPs that defined “complex problems” as having several (health) problems that often interact in old age, complex problems are operationalized as a combination of functional, somatic (health and illness), psychological and/or social problems (Blom *et al.*, 2015).

## Methods

For the EPISODE study we recruited persons aged ≥ 75 years from the ISCOPE (Integrated Systematic Care for Older PEople) study. The ISCOPE study aims to assess the effectiveness and cost-effectiveness of proactive, goal-oriented, integrated care in general practice with regard to the functioning of older people. It operationalizes care by developing an integrative plan for older persons with complex problems (Blom *et al.*, 2015). Community-living people aged ≥ 75 years received a short postal screening questionnaire covering information about their somatic, functional, mental, and social functioning. Older persons were visited at home to obtain data on social and demographic characteristics, and to administer additional questionnaires like the 15-item version Geriatric Depression Scale (GDS-15) (Sheikh and Yesavage, 1986).

Participants in the ISCOPE study were eligible for EPISODE if they gave a positive answer to the screening question: “*Have you recently felt downhearted and blue*?.” This is translated in the Dutch language as “*Heeft u zich de laatste tijd somber of neerslachtig gevoeld?*.” The word “somber,” often used by participants themselves, can also be translated as “low mood.” Purposeful sampling was applied in order to include an equal number of men and women. Participants differed regarding their residential arrangement, social situation (e.g. participants who were widowed, divorced or married) and their physical condition (e.g. participants with pain, a chronic disease, or multi-morbidity). Eligible participants were sent an invitation letter and a summary of the study, followed by a telephone call from a member of the research team.

We conducted two interviews, in a period of approximately six months, in order to provide participants with sufficient time to elaborate on the causes, solutions, and experience of their low mood or depressive feelings, as well as their perceptions about support and available interventions. Participants were informed about the explorative aim of the study.

### Structure of the interviews

Since all participants had indicated on the screening list that they (sometimes) had depressive feelings, the starting point of the first interview was to ask: “*You indicated that you have experienced a low mood [felt downhearted and blue] lately. Can you tell us why? Can you elaborate on this?*” These and additional questions enabled the participants to talk freely about their feelings and perceptions.

In the second interview, the findings from the first interview were summarized and participants were asked to reflect on these. We asked whether low mood was still experienced in the same way, or had changed. Then, individual needs and perceptions on support were assessed with questions such as: “*Are you satisfied with the way you feel? Can you think of certain aspects you would like to improve?*.”

Interviews were conducted by a medical anthropologist with experience in qualitative research among older people (MF); an anthropologist with experience in qualitative research (GG); and a GP with experience in qualitative research on depressive symptoms in older people (GW). As a team, the researchers received training in the use of Atlas ti, a qualitative data analysis program.

The interviews lasted about 1.5 h each and were conducted in the participant's home. Interviews were recorded and transcribed verbatim.

### Data analysis

Analysis of the data was based on an inductive approach according to the principles of grounded theory (Strauss and Corbin, 1998). Thematic content analysis was performed with Atlas ti. A coding scheme was developed and tested in an iterative fashion. Interview transcripts were coded independently by three researchers (MF, GG, GW); these codes were compared and any discrepancies were resolved through discussion and amendment. The codes were grouped into themes like causes, experiences of low mood and depressive feelings, coping, influencing factors, and needs and perceptions about interventions. Themes and findings were discussed with other senior researchers and led to new insights. For example, coping was initially split into two codes (coping and non-coping). However, discussion on respondents refraining from specific coping activities led to the addition of the theme of “risk” as assessed by respondents.

## Results

We aimed to include 40 older persons, with 10 persons in each group (men and women, with and without complex problems). For this, we contacted 55 persons from the former ISCOPE study before reaching a total of 40 persons who agreed to participate. Despite a MMSE score of 24, two participants were excluded after the first interview because of severe memory problems and Alzheimer's disease. The remaining 38 older persons (aged 77–90 years) with low mood (with and without complex problems) were further interviewed about their perceptions.

### Non-response

Of the 15 persons that chose not to participate, 9 had complex problems. The reasons to refuse participation in EPISODE were (i) participation in the ISCOPE study was considered to be enough, (ii) recent loss of a spouse, (iii) concerns about opening up old wounds, (iv) not wanting to talk about grief/sadness or private matters like marriage, or (v) hearing loss that would hinder the interviews.

### Demographic characteristics

The participants lived in seven different urban/rural communities in the western part of the Netherlands, and were registered in 16 different general practices. Table 1 presents the characteristics of the 38 study participants, as derived from the ISCOPE study.

**Table 1.** Demographic and clinical characteristics of the interviewed persons (*n* = 38)

*Demographic characteristics*		
Mean age in years (SD)	81.8	(3.8)
Women, n (%)	19	(50)
Living independently, n (%)	38	(100.0)
Low income *, n (%)	3	(7.9)
*Clinical characteristics*		
> 4 medications, n (%)	29	(76.3)
Multimorbidity		
> 2 diseases, n (%)	28	(73.7)
> 4 diseases, n (%)	18	(47.4)

*Low income: income from social security benefits only.

SD = standard deviation.

### Experience of low mood and depressive feelings

Although all participants indicated depressive symptoms in the screening, they did not always perceive this as a sign of depression. Most participants expressed feelings of “*somberheid*” which is best translated as “low mood” or “gloominess.” The way participants described this low mood ranged from experiencing a low mood incidentally (e.g. when reading a sad newspaper article) to experiencing depressive feelings every day and to the extent that they negatively influenced their overall well-being. Depressive feelings were sometimes perceived as an unavoidable and “appropriate” consequence of disruptive life events, such as the loss of a spouse or serious illness of a child. Six participants had been diagnosed with depression earlier in their life.

Participants defined a depression as a state of mind that was “out of their control” and could even be dangerous, e.g. leading to suicide, something the respondents did not identify with. Despite a low mood, most participants portrayed themselves as having positive characteristics and had a positive self-image.

“I think a depression means you can only think about negative things - but I don't have a negative attitude; there are lots of positive things, like the social contacts and visits from the family.” (Woman, no complex problems)

To their surprise, two participants had recently been diagnosed with a mild depression after they consulted their GP for memory complaints.

### Explanatory ideas for low mood and depressive feelings

Most participants gave a multi-dimensional explanation for their low mood or depressive feelings.

Social factors contributed for a major part to depressive feelings, e.g. loss of a spouse, feelings of loneliness, unhappiness in marriage, terminal illness of a child, conflict between their children, or loss of contact with children and/or grandchildren due to conflicts:

“I do not understand my wife, and she doesn't understand me. It's reciprocal. We live without any real contact.” (Man, complex problems)

“I try to make contact with my grandchildren. However, every time I ring them up, they [daughters] put the phone down.” (Man, no complex problems)

Physical and mental problems (such as physical limitations, memory complaints, chronic pain, sleep deficiency or fatigue) were perceived as influencing factors for a low mood. One woman (with complex problems) explained: “*It's difficult because I always suffer from pain. When I want to go out but can't because of the pain, I get depressed*.” Pain, or the use of diuretics, was also often a major factor in sleep disturbance.

Old age itself was another explanation given for the depressive feelings. A woman (without complex problems) said, “*Life is not as much fun as it used to be*.” Several participants were disappointed with this phase of their lives. They had expected a pleasant and quiet old age with an opportunity to enjoy life but, instead, had to deal with various problems and limitations. Some men were disappointed about their lives as a whole, or regretted decisions made earlier in life, e.g. their marriage.

Some participants mentioned anxiety and uncertainty about the future. They feared that their physical condition might decrease to such an extent that it might hamper solving conflicts and restoring lost social relationships before their death, or might interfere with their care-giving role as partner or parent. One woman (with complex problems) who still provided a lot of informal care said:

“When you get older, your resilience decreases. Two years ago, when my son had his heart operation, I could walk and visit him in the hospital. But at the moment I can't - and putting up with problems is getting more difficult. Mother isn't able to provide support any more. I’m afraid that I’ll lose control, that I’ll slip away.”

Individual character traits were sometimes mentioned as a cause for low mood. Some participants portrayed themselves as being different from others, being pessimistic or too perfectionistic with regard to their own social and cognitive functioning. Others referred to a life history of depressive feelings or a major depression in the past. One person had earlier been diagnosed with posttraumatic stress syndrome.

A few participants could not explain their low mood, mentioning that there was no specific reason.

### Ways of coping

A positive screening score on depressive symptoms did not necessarily mean that participants also expressed a need for professional support. Participants perceived dealing with low feelings as their individual responsibility. With respect to coping, they referred to their capability to solve the cause(s) or to accept the fact that low feelings would be part of their current lives. As one participant stated:

“In case of a low mood you have to seek the solutions yourself, or you have to learn to live with it - but for a major depression there are [professional] resources.” (Man, complex problems)

Individual coping strategies were directed at maintaining a balance and preserving a feeling of mastery and sense of continuity. These strategies could be divided into cognitive, social and practical strategies.

The cognitive strategies encompassed activities directed at fighting, accepting, or giving in to depressive feelings. Examples are: keeping up appearances for outsiders; a selective comparison with peers who are worse off; putting problems into perspective and a positive re-appraisal of the situation; making plans for the future and making a new start:

“I think ‘Okay. I can still walk, I see things, I’m able to use my hands and my mind is still good. I’m going to do things.’ I’m considering buying a new Apple computer, although the old one is still okay.” (Man, no complex problems)

Another strategy used was interrupting negative thoughts by, e.g. listening to music, reading, or walking the dog, or to give in to a low mood at certain moments, e.g. by consoling oneself with chocolate, a visit to the hairdresser, or buying something nice:

“When I feel down, I sit near the birdcage and talk to them [parakeets] - and then I make a cup of tea and have some chocolate.” (Woman, complex problems)

Avoidance of stress was also a deliberate choice in trying to preserve a balance and trying to master difficult situations, as one participant said:

“In the evening I turn off this mobile phone. I do have another one and that one is on, but she [alcoholic daughter] doesn't know the number. So my family and sons are able to contact me in the evening, but she is not.” (Man, complex problems)

Social strategies included increasing pleasant activities or hobby's with others, traveling, talking with someone, writing a letter, caring for others, doing voluntary work or problem-solving by discussing sensitive topics with children, as illustrated by a woman who decided to stay in her home against the will of her children:

“Well, it wasn't easy, I had to fight for it [the final decision not to move to a home for the elderly]. They didn't get it. They said: ‘But you can meet nice people and you can get together for a cup of coffee. There are so many advantages.’ I said: ‘I know, but that is outweighed by my feelings - I want to stay here and die here.’ So I had to fight for it.” (Woman, complex problems)

Inherent in the strategies was the assessment of possible risks, as participants were aware that it was sometimes better/necessary to refrain from certain activities, e.g. not talking with certain persons or about certain topics, in order to avoid conflict, as illustrated by one participant who for several years tried to restore the relationship between his three children, without interference of a third person:

“If an outsider would try to help I am sure it would make matters even worse. They would say: ‘Mind your own business.’ [. . .] because some friends and family contacted them earlier on - but this turned out negatively. That's why I’m afraid.” (Man, complex problems)

Practical strategies included self-medication, physical exercises, relaxation exercises, or consulting a GP for chronic pain or memory complaints. Other examples were seeking practical solutions for feelings of fear or anxiety, e.g. installing extra door locks in case of fear of burglary.

In dealing with their low mood or depressive feelings, participants held a clear view about their own role in coping. Several patterns emerged from the data:

Mastery. “I’ll seek a solution myself - when I think it's necessary.”

Of the 38 participants, 19 felt they were sufficiently capable of coping. They stated that in their current situation they did not need professional support and trusted their self-efficacy or the available social support. This was mainly the case for women without complex problems, but also for some participants with complex problems, as one man said: “*I’ve been through a lot. I’m 77 years old now [. .] I’ll manage*.”

With regard to mastery, participants referred to their knowledge about the possibilities for support and former experiences, and sometimes to their goals/plans for the future.

Statements about mastery also showed self-management with regard to medication (e.g. buying over-the-counter medicines, or autonomously changing the medication dose) and decisions *not* to comply with the medical advice from the GP or specialist. In conclusion, participants with a feeling of mastery experienced a satisfactory feeling of “being in control.”

Participants with a strong sense of mastery were convinced they would receive sufficient professional help should they ask for it. Risks played no important role in their perception of the future.

Acceptance. “I accept it the way it is - but there is something that no longer fits.”

Eight participants indicated that their problem could not be solved, e.g. in case of an unsatisfactory relationship with the partner or children. They tried to accept their current situation, to distract their negative thoughts, and to focus on positive aspects. Some even remembered a specific moment or event that made them switch their perception, as stated by this woman:

“I had the feeling that everyone was angry with me - eventually it became an obsession. Then one night I got up and I sat at this table. Then I thought: No, I won't let it influence my life any more. I’ll put them [worries] in a box and throw away the key.” (Woman, complex problems)

This attitude resulted in an acceptance of the situation, instead of continuing to worry and seek solutions.

Ambivalence. “Maybe you’ll meet someone. On the other hand, you might not meet someone you can get along with - I won't run that risk!”

Seven participants mentioned that they were not content with their current situation. They wanted things to change and mentioned possible solutions; however, they were unable to bring about these changes themselves. With regard to the solutions, they weighed the “pros and cons,” like fear for discontinuity, as one man (no complex problems) explained:

“I have my own schedule [. . .]. If someone disturbs this programme, I’m upset for days.”

Moreover, they feared several “risks” such as: not being in control and unable to foresee the consequences of support and/or interventions (especially participants with complex problems found this difficult); getting feelings of false hope; a further decrease of self-esteem (e.g. because of memory loss or inability to concentrate in social gatherings); and being worse off and unable to reverse certain decisions once made. The statements of one woman illustrate this topic. She had always been proud of being a “proper housewife” but due to physical limitations she was no longer able to fulfill this role. In addition, she worried about the strain this puts on her husband who had to perform the household activities and, at the same time, was unable to adhere to her norms about the way the tasks should be carried out. She doubted whether professional support would be a good solution:

“It could be a relief, but then again it might bring new annoyances.” (Woman, complex problems)

The GP was mainly associated with somatic problems and the prescription of medication. With regard to depressive feelings, participants referred to the expected (in)ability of their GP to solve the cause.

“Should I take medication? I don't want to take pills, at least not for this problem. She [GP] won't be able to solve the problem [conflict with daughters].” (Man, no complex problems)

Some participants stated that they did not want to run the risk that discussing low feelings or other problems would result in a change of medication or receiving professional advice that they could not or would not follow. Most participants were also afraid to be perceived by the GP as “whining” about aspects that one should accept. These fears made several participants hesitant towards changes that might improve their situation.

Some problems needed interventions on multiple domains of social, physical and cognitive functioning at the same time. For some participants the balance was already so fragile that they feared each change; they indicated that they felt tired and had little energy left.

Need for support. “I would like things to change, but what can I do? What can be done? I really don't know.”

Four participants mentioned that they needed specific professional care. Although they invested much time and effort in coping, this was no longer sufficient; a feeling of depression, lack of hope, and inability to control the problems dominated their conversations.

Although they were already consulting the GP for physical problems, not all of them reported their psychological distress to the GP. Participants expected GPs to be well informed about the state of affairs of their patients and take the initiative in discussing depressive symptoms when considered necessary. Participants expressed needs about adequate pain medication, assistance in finding a new home, re-examination of anti-depressants, referral to a psychologist, and a supportive attitude in case of a degenerative disease:

“All I want is that he [GP] takes a closer look at the medication [for pain] But he said: ‘Older people should not have too much medication because they have an increased risk of falling.’ (Woman, complex problems)

It was unclear for some participants who could improve their situation and in what way, e.g. in case of declining health combined with a lack of financial and/or social resources. For three of the participants with complex problems, financial problems limited their coping abilities (e.g. moving to another house).

Participants with a history of depressive feelings and complex problems feared false hopes, and some had lost confidence in professional solutions. One woman with persistent depressive feelings, sleeping problems, and feelings of loneliness, who had received psychotherapy in the past and participated in two courses directed at dealing with these problems, said:

“The GP also said that talking to welfare workers would be a solution. But I’ve had that before. They were nice to talk to, but what can they do? You can tell your story in detail and then that's the end. It is like when I was in . . .[name institution].” (Woman, complex problems)

Furthermore, whether or not to discuss a low mood with the GP was influenced by the communicative skills of their physician or the doctor-patient relationship.

### Dynamics of coping and expressing needs for support

Coping with regard to low mood and depressive feelings appeared to be dynamic, i.e. to change over time. In the present study, the first interview sometimes led to decisions and practical solutions, or cognitive re-appraisal by the participants. In the second interview, participants mentioned the recent changes in their situation and how these had influenced their mood. Positive changes could be, for example, an improved physical condition, new medication, or a positive conversation with children about a difficult topic. The following example shows how a favorable outcome on a follow-up visit for cancer had a positive effect on low mood and coping:

“These low feelings are much less. I’m distancing myself from the illness trajectory; the prospects to live have increased. Last time, the diagnosis in the hospital was that the cancer was completely gone. The radiologist told me: ‘Just continue with your life. [. . .]’ That brightens you up completely. I feel a lot better. I have started to paint again - which is very nice. We’ve also bought new things, like a television and an iPad for playing games.” (Man, complex problems)

Negative changes included increasing pain, fatigue, a fall, a disappointing outcome of a discussion, or a worsening condition of a child. These developments led to new problems, specific needs for support, or an intensified effort to distract negative thoughts. A few participants discussed their mood with the GP and accepted the prescription for antidepressants.

In general, participants with a sense of mastery and positive experiences with support were more positive about taking the initiative to bring up the subject.

## Discussion

In the present study, participants with depressive feelings (identified via screening), with and without complex problems, were engaged in coping and solution-seeking behavior. This almost invisible “work” (Corbin and Strauss, 1988) was directed at trying to master (complex) problems, and sometimes simultaneously accepting or fighting depressive feelings. The coping strategies included elements of effective interventions, e.g. a positive re-appraisal of their situation.

Most factors that influence the perspectives of individuals in general practice on depressive feelings have been described previously, such as self-responsibility in dealing with low mood or depressive feelings (e.g. Switzer *et al.*, 2006; Wittkampf *et al.*, 2008; Prins *et al.*, 2011, van der Weele, *et al.*, 2012). In the present study, we found that this was equally important for participants with and without complex problems. Their coping was related to perceptions about the causes, rather than the consequences of depressive feelings, and most participants did not expect their problems to be “solved” by a professional intervention.

As also reported by others (Wittink *et al.*, 2006), most participants believed that their GP was able to recognize and discuss their depression, when deemed relevant from a professional perspective, because the GP knew about their circumstances.

All those who expressed a need for professional support were participants with complex problems who felt that their own ability to cope was no longer sufficient.

For the interpretation of the findings it is important to describe whether participants in this study had minor or significant depressive symptoms. Because this research was part of the larger ISCOPE study we were able to extract the GDS scores. The individual scores ranged from 0–9, with one exception for a participant diagnosed with PTSS with a GDS score of 13. Several participants reported that they had received treatment for depressive feelings in the past, or currently used medication. Especially for participants with low expectations about professional support because of negative experiences in the past, coping has to be viewed from a life-span perspective.

Statements of mastery were mentioned mostly by women without complex problems. Among participants with greater morbidity we found many differences in statements about depression and coping. This may be explained by the fact that high morbidity was not always perceived as a cause for depressive feelings and was (to a certain extent) accepted.

Men with complex problems often referred to problems in their social relationships and were trying to accept this, or find means to resolve these problems themselves. Social roles related to parenthood proved to be important for both men and women. The negative influence of the loss of social roles on depressive feelings is also documented among patients with multi-morbidity (Stanners *et al.*, 2014).

Our findings suggest that older people with complex problems, although generally satisfied with their coping ability, also refer to limited means for coping due to high morbidity, fatigue, and problems with concentration or pain. A recent study found that pain is more influential than chronic disease in relation to depressive symptoms (Gerrits *et al.*, 2014).

Although the concerns of depressed patients about possible harm has been reported with regard to the use of antidepressants and medication (van Schaik *et al.*, 2004; Burroughs *et al.*, 2006), it is useful to extend this notion of harm to perceived disadvantages with regard to professional interventions in general.

Interventions may lead to feelings of being “out of control” and discontinuity, thereby disturbing the individual's fragile balance. The perceptions of older persons regarding the risks involved may influence the acceptability of proposed treatments (Chew-Graham *et al.*, 2012) and explain why they refrain from certain interventions or fail to disclose depressive feelings to peers, family or professionals (Wittink *et al.*, 2006).

In accordance with Cornford *et al.* (2007), we propose that the coping techniques applied by patients to control their depressive feelings could be used as a basis for the GP to monitor patients’ self-efficacy and needs over time. Talking about low mood enables the physician to explore patients’ perceptions of (treatment) risks, to provide information and to discuss alternative options. The burden is on physicians to receive further training, or to refer to other health professionals or welfare workers who can address the patients’ problems and depressive feelings in an appropriate way. In addition, there is a need for older patients to be made aware of the legitimacy to consult their GP about low mood (Burroughs *et al.*, 2006) and to increase their knowledge about symptoms of depression.

### Study strengths

The present qualitative study was nested in a large population-based study which included measurements of functional, somatic, mental, and social health (Blom *et al.*, 2015). This provided the advantage of having sufficient eligible participants to include a sample with an optimal level of variety.

Conducting two interviews revealed the dynamics in the situation of the participants and, accordingly, the effects on their depressive feelings, needs and coping strategies.

### Study limitations

Although eight participants had a former diagnosis of depression, in this study we may have missed older people with a severe clinical depression because of selective non-response. Also, we are unable to generalize these results to older people with cognitive decline and depressive feelings, because such older persons were not included.

### Implications and conclusion

Older patients do not expect their GP to present easy solutions, but do expect the GP to play a role in guiding and providing a safety net for those with low mood or depressive feelings. The results indicate that it is relevant for GPs to take the initiative in raising the topic of low mood in old age. Instead of putting effort in trying to convince older people to participate in interventions, it may be more useful to monitor changes in low mood or depressive symptoms and to discuss whether patients perceive their coping as (still) being sufficient. This may be equally important for participants with only minor mood problems as they will receive useful information, as for patients with more serious depressive symptoms or a diagnosis of depression; also, discussing their needs and perception of treatment risks may increase trust in professionals and the acceptability of therapy. This approach can be tailored to the needs of older people and may improve the dialogue between patient and GP.

## Conflict of interest

None.

## Description of authors’ roles

Margaret von Faber: study concept and design, obtaining funding, acquisition of data, analysis and interpretation of data, preparation of the manuscript, final approval. Geertje van der Geest: acquisition of data, preparation of the manuscript, final approval. Gerda van der Weele: study concept and design, acquisition of data, preparation of the manuscript, final approval. Jeanet Blom: study concept and design, analysis and interpretation of data, preparation of the manuscript, final approval. Roos van der Mast: study concept and design, preparation of the manuscript, final approval. Ria Reis: study concept and design, preparation of the manuscript, final approval. Jacobijn Gussekloo: study concept and design, obtaining funding, acquisition of data, analysis and interpretation of data, preparation of the manuscript, final approval.

## Ethical approval

The study was approved in 2011 by the Medical Ethical committee of Leiden University Medical Center (Study no. P09.096). All participants gave written informed consent.

## References

[ref001] Beekman A. T., Penninx B. W., Deeg D. J., de Beurs E., Geerling S. W. and van Tilburg W. (2002). The impact of depression on the well-being, disability and use of services in older adults: a longitudinal perspective. Acta Psychiatrica Scandinavica, 105, 20–27. 12086221 10.1034/j.1600-0447.2002.10078.x

[ref002] Beljouw I. M. J. et al. (2014). Implementing an outreaching, preference-led stepped care intervention programme to reduce late life depressive symptoms: results of a mixed methods study. Implementation Science, 9, 107–119. doi:10.1186/s13012-014-0107-y. 25163984 PMC4156632

[ref003] Blom J. W. et al. (2015). Effectiveness and cost-effectiveness of a proactive, goal-oriented, integrated care model in general practice for older people. A cluster randomized controlled trial: integrated systematic care for older people — the ISCOPE study*. Age and Ageing*, Accepted for publication. 10.1093/ageing/afv174 PMC4711660 26764392

[ref004] Burroughs H., Lovell K., Morley M., Baldwin R., Burns A. and Chew-Graham C. (2006). “Justifiable depression”: how primary care professionals and patients view late-life depression? A qualitative study. Family Practice, 23, 369–377. 16476699 10.1093/fampra/cmi115

[ref005] Chew-Graham C. et al. (2012). Why may older people with depression not present to primary care? Messages from secondary analysis of qualitative data. Health and Social Care in the Community, 20, 52–60. 21749528 10.1111/j.1365-2524.2011.01015.x

[ref006] Comijs H. C. et al. (2011). The Netherlands study of depression in older persons (NESDO); a prospective cohort study. BMC Research Notes, 4, 524. doi:10.1186/1756-0500-4-524. 22142532 PMC3278450

[ref007] Conner K. O. et al. (2010). Attitudes and beliefs about mental health among African-American older adults suffering from depression. Journal of Aging Studies, 24, 266–277. 21423819 10.1016/j.jaging.2010.05.007 PMC3060786

[ref008] Corbin J. M, and Strauss A. (1988). Unending Work and Care: Managing Chronic Illness at Home. San Francisco: Jossey-Bass.

[ref009] Cornford C. S., Hill A. and Reilly J. (2007). How patients with depressive symptoms view their condition: a qualitative study. Family Practice, 24, 358–364. 17630269 10.1093/fampra/cmm032

[ref010] Cuijpers P., Van Straten A., van Schaik A. and Andersson A. (2009). Psychological treatment of depression in primary care: a meta-analysis. British Journal of General Practice, 59, 51–60. doi:10.3399/bjgp09×395139. 19192368 PMC2629842

[ref011] Gerrits M. J. G., van Oppen P., Leone S. S., van Marwijk H. W., van der Horst H. E. and Penninx B. W. (2014). Pain, not chronic disease, is associated with the recurrence of depressive and anxiety disorders. BMC Psychiatry, 14, 187. doi:10.1186/1471-244X-14-187. 24965597 PMC4090396

[ref012] Joo J. H., Wittink M. and Dahlberg B. (2011). Shared conceptualizations and divergent experiences of counseling among African American and white older adults. Qualitative Health Research, 21, 1065–1074. 21464469 10.1177/1049732311404247 PMC6588405

[ref013] Lawrence V., Banerjee S., Bhugra D., Sangha K., Turner S. and Murray J. (2006). Coping with depression in later life: a qualitative study of help-seeking in three ethnic groups. Psychological Medicine, 36, 1375–1383. 16854247 10.1017/S0033291706008117

[ref014] Lyness J. M., Pin Q., Tang W., Tu X. and Conwelll Y. (2009). Risk for depression onset in primary care elderly patients: potential targets for preventive interventions. American Journal of Psychiatry, 166, 1375–1383. doi:10.1176/appi.ajp.2009.08101489. 19833788 PMC2982671

[ref015] Mitchel A. J., Rao S. and Vaze A. (2011). International comparison of clinicians’ ability to identify depression in primary care: meta-analysis and meta-regression of predictors. British Journal of General Practice, 61, 72–80. doi:10.3399/bjgp11×556227. 21276327 PMC3026173

[ref016] Murray J., Banerjee S., Byng R., Tylee A., Bugra D. and MacDonald A. (2006). Primary care professionals’ perceptions of depression in older people: a qualitative study. Social Science & Medicine, 63, 1363–1373. 16698157 10.1016/j.socscimed.2006.03.037

[ref017] Prins M. A., Verhaak P. F., Bensing J. M. and van der Meer K. (2008). Health beliefs and perceived need for mental health care of anxiety and depression-The patients’ perspective explored. Clinical Psychology Review, 28, 1038–1058. doi:10.1016/j.cpr.2008.02.009. 18420323

[ref018] Prins M. A. et al. (2011). Perceived need for mental health care and barriers to care in the Netherlands and Australia. Social Psychiatry and Psychiatric Epidemiology, 46, 1033–1044. doi:10.1007/s00127-010-0266-3. 20686887 PMC3173635

[ref019] Romijn G., Ruiter M. and Smit F. (2007). Meer Effect met Depressiepreventie? Strategieën voor Publieksvoorlichting, Vroegherkenning en Terugvalpreventie. Utrecht: Trimbos-Institute. Dutch.

[ref020] Sheikh J. A. and Yesavage J. A. (1986). Geriatric depression scale (GDS): recent findings and development of a shorter version In L. T. Brink (ed.), Clinical Gerontology; a Guide to Assessment and Intervention (pp. 165–173). New York: Howarth Press.

[ref021] Stanners M. N., Barton C. A., Shakib S. and Winefield H. R. (2014). Depression diagnosis and treatment amongst multimorbid patients: a thematic analysis. BMC Family Practice, 15, 124. doi:10.1186/1471-2296-15-124. 24947875 PMC4074384

[ref022] Stek M. L., Gussekloo J., Beekman A. T. F., van Tilburg W. and Westendorp R. G. J. (2004). Prevalence, correlates and recognition of depression in the oldest old: the Leiden 85-plus study. Journal of Affective Disorders, 78, 193–200. 15013243 10.1016/S0165-0327(02)00310-5

[ref023] Strauss A. and Corbin J. (1998). Basics of Qualitative Research: Techniques and Procedures for Developing Grounded Theory. London: Sage.

[ref024] Switzer J. F., Wittink M. N., Karsch B. B. and Barg F. K. (2006). “Pull yourself up by your bootstraps”: a response to depression in older adults. Qualitative Health Research, 16, 1207–1216. 17038753 10.1177/1049732306290148 PMC2782756

[ref025] Van der Weele G. M. et al. (2012). Response to an unsolicited intervention offer to persons aged ≥ 75 years after screening positive for depressive symptoms: a qualitative study. International Psychogeriatrics, 24, 270–277. doi:10.1017/S1041610211001530. 21846427

[ref026] Van Schaik D. J. et al. (2004). Patients’ preferences in the treatment of depressive disorder in primary care. General Hospital Psychiatry, 26, 184–189. 15121346 10.1016/j.genhosppsych.2003.12.001

[ref027] Wittink M. N., Barg F. K. and Gallo J. J. (2006). Unwritten rules of talking to doctors about depression: integrating qualitative and quantitative methods. Annals of Family Medicine, 4, 302–309. 16868233 10.1370/afm.558 PMC1522158

[ref028] Wittkampf K. A., van Zwieten M., Smits F. T., Schene A. H., Huyser J. and van Weert H. C. (2008). Patients’ view on screening for depression in general practice. Family Practice, 25, 438–444. doi:10.1093/fampra/cmn057. 18836095

